# A conserved arginine residue is critical for stabilizing the N2 FeS cluster in mitochondrial complex I

**DOI:** 10.1016/j.jbc.2021.100474

**Published:** 2021-02-26

**Authors:** Mikhail A. Hameedi, Daniel N. Grba, Katherine H. Richardson, Andrew J.Y. Jones, Wei Song, Maxie M. Roessler, John J. Wright, Judy Hirst

**Affiliations:** 1 Medical Research Council Mitochondrial Biology Unit, University of Cambridge, Cambridge, UK; 2 School of Biological and Chemical Sciences, Queen Mary University of London, London, UK; 3 Department of Chemistry, Imperial College London, Molecular Sciences Research Hub, White City Campus, London, UK

**Keywords:** complex I, cryo-electron microscopy, dimethyl-arginine, electron paramagnetic resonance (EPR), iron–sulfur cluster, NADH:ubiquinone oxidoreductase, *Yarrowia lipolytica*, BN-PAGE, blue native polyacrylamide gel electrophoresis, complex I, NADH:ubiquinone oxidoreductase, cryo-EM, electron cryomicroscopy, DSF, differential scanning fluorimetry, EPR, electron paramagnetic resonance, FeS, iron–sulfur, NDH2, type II NADH:ubiquinone oxidoreductase, ROS, reactive oxygen species

## Abstract

Respiratory complex I (NADH:ubiquinone oxidoreductase), the first enzyme of the electron-transport chain, captures the free energy released by NADH oxidation and ubiquinone reduction to translocate protons across an energy-transducing membrane and drive ATP synthesis during oxidative phosphorylation. The cofactor that transfers the electrons directly to ubiquinone is an iron–sulfur cluster (N2) located in the NDUFS2/NUCM subunit. A nearby arginine residue (R121), which forms part of the second coordination sphere of the N2 cluster, is known to be posttranslationally dimethylated but its functional and structural significance are not known. Here, we show that mutations of this arginine residue (R121M/K) abolish the quinone-reductase activity, concomitant with disappearance of the N2 signature from the electron paramagnetic resonance (EPR) spectrum. Analysis of the cryo-EM structure of NDUFS2-R121M complex I at 3.7 Å resolution identified the absence of the cubane N2 cluster as the cause of the dysfunction, within an otherwise intact enzyme. The mutation further induced localized disorder in nearby elements of the quinone-binding site, consistent with the close connections between the cluster and substrate-binding regions. Our results demonstrate that R121 is required for the formation and/or stability of the N2 cluster and highlight the importance of structural analyses for mechanistic interpretation of biochemical and spectroscopic data on complex I variants.

Mitochondrial complex I (NADH:ubiquinone oxidoreductase) is a key energy-transducing enzyme in oxidative phosphorylation (1, 2, 3). This large (∼1 MDa) and intricate enzyme catalyzes the oxidation of NADH and reduction of ubiquinone, capturing the free energy released to drive protons across the inner mitochondrial membrane, contributing to the proton-motive force and sustaining ATP synthesis. It is also crucial for regenerating NAD^+^ to maintain tricarboxylic acid cycle function and the β-oxidation of fatty acids. The central metabolic role of complex I, combined with its propensity to generate reactive oxygen species (ROS), makes complex I dysfunction a key contributor to a substantial array of metabolic and neurodegenerative disorders (4, 5). Recent advances in single-particle electron cryomicroscopy (cryo-EM) have broadened the scope for investigation of complex I (2, 6, 7) and enabled detailed structural analyses of defined biochemical states (8, 9, 10, 11). Furthermore, the application of high-resolution cryo-EM to genetically tractable model organisms provides a platform to investigate the structural effects of debilitating mutations of complex I, in combination with robust functional examination. *Yarrowia lipolytica* is one such model organism that is well established for studies of complex I (12, 13, 14, 15). Complex I from *Y. lipolytica* contains 43 subunits, of which 14 are the core subunits required for catalytic function and 29 are supernumerary subunits with varied roles in assembly, stability, regulation, or independent metabolic function (1, 16, 17, 18). The structure of *Y. lipolytica* complex I has been resolved by X-ray crystallography and cryo-EM (10, 19, 20), with the recent 2.7 Å resolution wild-type structure (21) providing an excellent basis for comparison with specific mutant variants.

During catalysis, electrons from NADH tunnel through the hydrophilic domain of complex I to the ubiquinone-binding site (∼20 Å above the membrane plane) *via* a chain of iron–sulfur (FeS) clusters. The terminal cluster of the FeS relay is a [4Fe–4S] cluster, referred to as cluster N2 that is located at the interface of the NDUFS2 (NUCM) and NDUFS7 (NUKM) subunits. Note that we use the nomenclature for human complex I (for example, NDUFS2) for simplicity, rather than that specific to *Y. lipolytica* (for example, NUCM). Cluster N2 is positioned ∼12 Å above the quinone-binding site and ligated by four cysteine residues in NDUFS7, including an unusual, strained tandem cysteine pair (C85 and C86 in *Y. lipolytica*) (22). It donates electrons directly to ubiquinone and has received considerable attention due to its unique properties among the complex I FeS clusters, particularly its relatively high, pH-dependent reduction potential (23). Several mutations in the second coordination sphere of N2 have been shown to impact the quinone reductase activity and inhibitor binding of complex I (24, 25, 26, 27, 28, 29, 30, 31). One such residue, NDUFS2-H226 forms a hydrogen bond to the N2 cluster, an interaction that has a strong impact in tuning its reduction potential (26, 32) but has little effect on the functional capacity of the enzyme. Surprisingly, other local mutations have been shown to deplete the N2 electron paramagnetic resonance (EPR) signal while retaining a degree of quinone reductase activity. Conservative mutations in NDUFS2-R141 have been reported to produce partially active (∼40–50% of parental strain) complex I that appears to lack an N2 cluster, indicating the possibility of quinone reduction in the absence of a reduced N2 cluster by an unknown mechanism (25, 33). Further interpretations of specific mutations in the second coordination sphere of N2 that impact enzyme turnover have been limited by the requirement for high-resolution structural data to pinpoint their inhibitory effects.

A highly conserved stretch of residues in the N terminus of the NDUFS2 subunit has previously been identified as an important structural element in the stability of complex I in *Y. lipolytica* (24, 27). This “HRGXE-motif,” which is partially conserved in [NiFe] hydrogenases (24, 34), is located close to the N2 cluster, where several of the motif residues are involved in the formation of its secondary coordination sphere (24, 27). The arginine residue (NDUFS2-R121) in this motif is particularly intriguing, owing to its proximity to the N2 cluster (3.7 Å) and its unusual posttranslational modification: R121 has been shown to be the target of symmetrical ω-N^G^,N^G^ dimethylation on its guanidino group (35). Conservation of this posttranslational modification across mammalian (35), yeast (*Pichia pastoris* (35) and *Y. lipolytica* (21)), and bacterial (*Paracoccus denitrificans* (35)) complex I suggests that the modification is functionally significant, and suppression of NDUFAF7 expression, the methyltransferase responsible for dimethylation of R121 in humans, results in impaired complex I assembly in cultured human cells (35, 36). It has been suggested that dimethylation of this arginine residue is required for the formation of a 400 kDa assembly intermediate (36, 37). Furthermore, the hydrophobicity conferred by the two methyl groups may alter the reduction potential of the N2 cluster, decreasing the hydrogen-bonding capacity of the arginine side chain (36). Mutations in this unusual dimethylated arginine have been associated with the neurological disorder Leigh syndrome, suggested to stem from an altered reduction potential of the N2 cluster leading to a disruption of electron transfer to ubiquinone (38, 39). The mechanisms by which R121 functions in either assembly or catalysis are yet unconfirmed; however, these studies highlight its unique role for complex I assembly/activity.

NDUFS2-R121 mutants have been reported to show similar hallmarks to the N2-deficient R141 mutants mentioned above, including a decrease in membrane complex I content as well as a severe ablation of quinone reductase activity in membranes (30). The relatively conservative substitution of R121 to lysine (R121K) markedly decreased both the complex I content in mitochondrial membranes (to ∼40% of wild-type) and the specific activity (dNADH:DQ) to 24% of the parental strain. Importantly, no complex-I-specific EPR spectrum could be distinguished from NADH-reduced mitochondrial membranes, an effect not observed in other HRGXE-motif mutants. Whether this effect was reflective of the low complex I content in the mitochondrial membranes or a diminished N2 signal directly was not established (30). While R121 is highly conserved across the majority of complex I species, it is replaced with a threonine residue in *Thermus thermophilus*, the first organism for which a crystal structure of complex I was obtained (22, 40). Interestingly, the canonical EPR signature of the reduced N2 cluster has not been observed in membrane particles or the hydrophilic domain of complex I from *T. thermophilus* upon reduction by NADH (41, 42). This has led to suggestions that the absence of R121 (or the presence of threonine) in the second coordination sphere of N2 in the *T. thermophilus* enzyme may either decrease the reduction potential of the cluster below that of NADH or alter its spin state such that the cluster is not detected under standard EPR measurement conditions (43). Determining the relationships between the dimethylated R121 residue and cluster N2 should give insights into the role of this residue, in catalysis and/or assembly.

Here, we used two variants of NDUFS2-R121 to investigate its functional and structural role in *Y. lipolytica* complex I. We combine biochemical characterization of R121 mutants with cryo-EM structural analysis to show that R121 mutants are capable of native-like assembly but suffer from an absolute loss of quinone reductase activity. EPR spectroscopy and examination of the local structure confirm the absence of a functional N2 cluster, with profound effects on the cysteine residues in the N2 primary coordination sphere, as well as critical ubiquinone-binding residues. Our data confirms that electron transfer to ubiquinone cannot occur in the absence of an N2 cluster, despite the otherwise native-like structural assembly, and suggests why R121 is so critical for the function of the complex.

## Results

### NDUFS2-R121 mutant strains produce intact complex I with impaired quinone reductase activity

**Figure 1:**
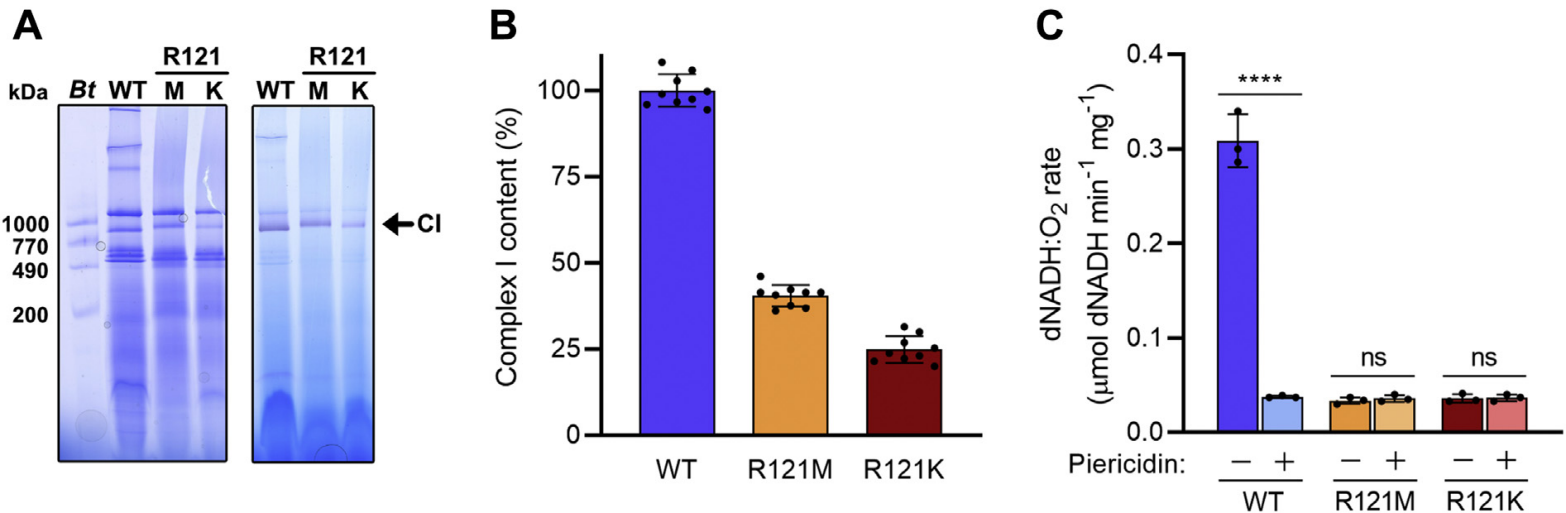
**Comparison of the R121M and R121K variants of complex I in mitochondrial membranes from *Y. lipolytica*.***A*, structural integrity of complex I demonstrated using BN-PAGE analysis with Coomassie blue staining (*left*) and in-gel NADH oxidation activity (*right*) of solubilized mitochondrial membranes. In total, 40 μg of total membrane protein (WT = GB10 complex I, R121M, R121K) was solubilized with DDM at a 2:1 weight ratio. A reference sample of bovine mitochondrial membranes (*Bt*) is given. NADH oxidation activities were detected using 0.5 mg ml^−1^ NBT and 150 μM NADH to identify complex I containing bands (62) (see Experimental procedures). The gels shown are representative of three BN-PAGE replicates. *B*, the amounts of R121M and R121K complex I present in the membranes were determined using the dNADH:APAD^+^ activity by comparison to the dNADH:APAD^+^ activity of the wild-type membranes (100%) (see Experimental procedures for assay details). Graphs show mean averages ± SD (n = 9) with individual data points in *black*. *C*, dNADH:O_2_ activities of wild-type, R121M and R121K membranes determined in the presence and absence of 1 μM piericidin A (see Experimental procedures for assay details). Graphs show mean averages ±SD (n = 3) with individual data points in *black*. Data were evaluated by one-way ANOVA with Tukey’s multiple comparisons correction (∗∗∗∗*p* < 0.0001). No significant differences were observed between piericidin A-inhibited WT and the R121 mutants (±piericidin A).

Two variants of NDUFS2-R121 were created by site-directed mutagenesis: the R121M and R121K variants. Previous work on the R121K variant reported depletion of the complex I content in *Y. lipolytica* membranes (30), suggesting that R121 mutants result in decreased complex I stability. Our initial characterization was therefore focused on establishing the structural integrity of both the membrane-bound and detergent-solubilized enzymes. Figure 1*A* shows a blue native (BN)-PAGE analysis of wild-type (we use the term wild-type (WT) to refer to the parental GB10 strain) and mutant *Y. lipolytica* membranes solubilized with *n*-dodecyl-β-D-maltoside (DDM). Complex-I-containing bands were identified by in-gel activity staining for NADH oxidation. In all cases, the BN-PAGE clearly shows a band at approximately 900 kDa that is positive for NADH oxidation activity, demonstrating that complex I is present and intact in membranes from both variants. The relative intensities of the bands for the R121M and R121K variants suggest a lower complex I content than for wild-type, most obviously for R121K. The decrease in complex I content was quantified using the rate of APAD^+^ reduction by dNADH, a specific substrate for the complex I flavin site, which is not expected to be affected by the mutations (Fig. 1*B*). dNADH:APAD^+^ activity indicated that the complex I contents were ∼40% and 25% for R121M and R121K, respectively, relative to wild-type, corroborating the observations from BN-PAGE. These results are in line with those previously obtained for R121K (30), where complex I content was seen to decrease to ∼40% of the wild-type. Therefore, although it is clear that mutations of R121 impair the assembly and/or stability of complex I, a level of intact enzyme is still present and available for study. The quinone reductase activities, measured with the complex I-specific substrate dNADH, showed extensive loss of the activity in both the R121M and R121K variants, in comparison to wild-type (Fig. 1*C*). The minor rates observed in the mutants were insensitive to piericidin A, a complex I inhibitor and Q analogue, clearly demonstrating a lack of any specific catalytic activity, despite the apparent structural integrity of the enzymes themselves.

**Figure 2:**
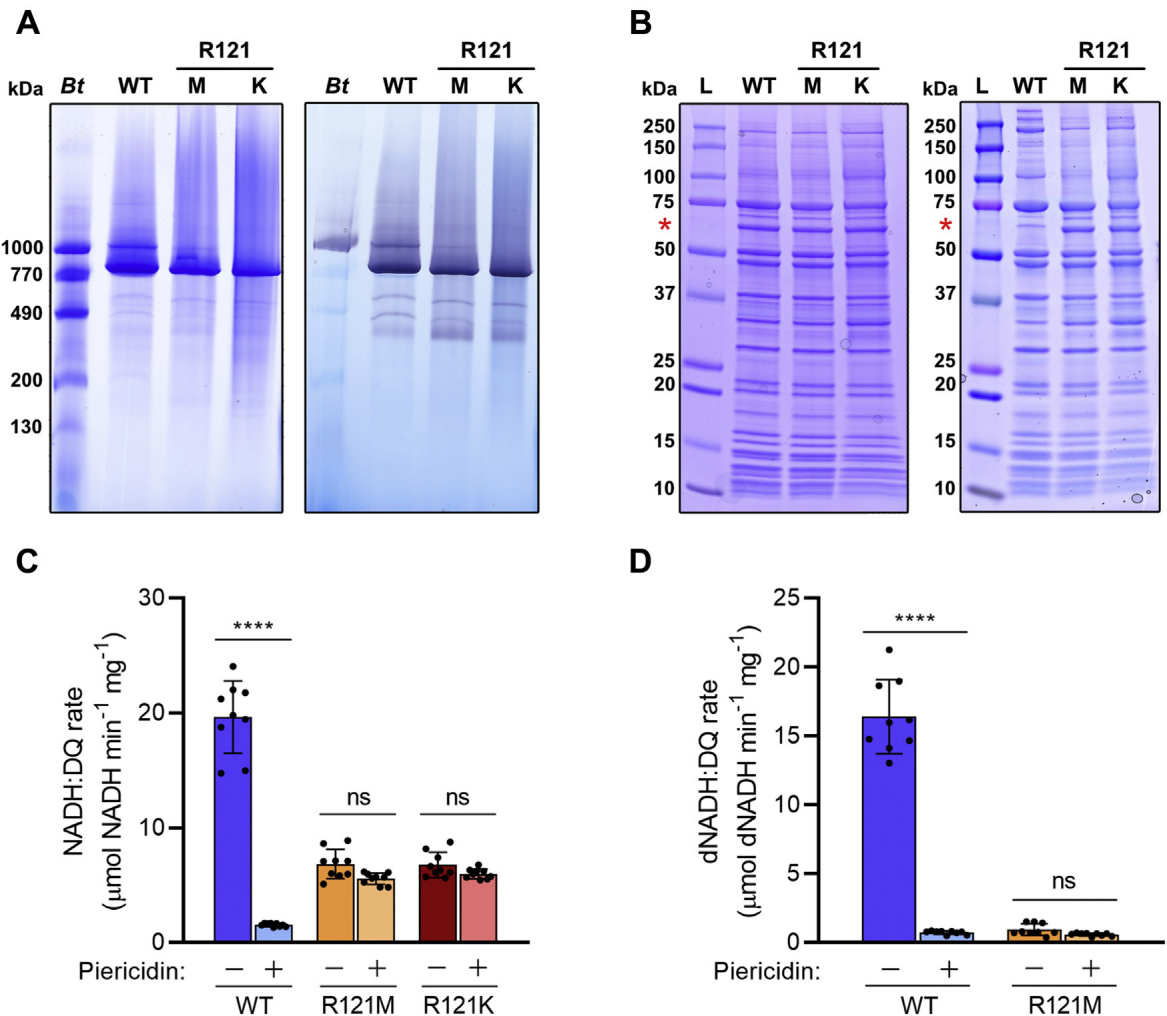
**Analysis of the composition and specific activity of the purified R121M and R121K complex I variants in comparison to the WT enzyme.***A*, BN-PAGE analysis with Coomassie staining (*left*) and in-gel NADH oxidation activity (*right*) of the purified enzymes. In total, 40 μg of each sample was loaded onto the gel and compared with a reference sample of bovine mitochondrial membranes (*Bt*). In-gel activities were performed as in Figure 1*A*. Gels are representative of three replicates. *B*, SDS-PAGE analysis of the purified wild-type and R121 variant complexes. In total, 20 μg of each sample was loaded and compared with a protein standard (L – see Experimental procedures). The *red asterisk* indicates a 60 kDa band corresponding to the small quinone reductase NDH2, not previously observed (44), which is copurified as a contaminant with *Y. lipolytica* complex I. The gel on the *right* shows a replicate gel highlighting the varying levels of NDH2 contamination observed in different wild-type preparations in comparison to the mutant samples where the contamination is consistently present. These are representative gels of three replicates. *C*, NADH:DQ activities in the presence and absence of 1 μM piericidin A (see Experimental procedures for assay details). *D*, dNADH:DQ activities in the presence and absence of 1 μM piericidin A (see Experimental procedures for assay details). All activity values are mean averages ± SD (n = 9) evaluated with one-way ANOVA with Tukey’s multiple comparisons correction (∗∗∗∗*p* < 0.0001). Individual data points are given in *black*.

To gain further insight into their stability, the R121 variants were purified from mitochondrial membranes and their functional capacities characterized. Both variants exhibited substantial levels of aggregation in the size-exclusion chromatographic traces. However, analyses of the monomeric peak fractions by BN-PAGE and SDS-PAGE revealed enzymes that closely match the wild-type enzyme. Both Coomassie and in-gel activity staining of BN-PAGE analyses (Fig. 2*A*) indicate a homogeneous preparation of intact complex I, with a functional flavin site. SDS-PAGE analyses revealed near-identical protein compositions in preparations of the wild-type and mutant enzymes (Fig. 2*B*). An additional band was present at ∼60 kDa that was not present in previously analyzed wild-type *Y. lipolytica* complex I (44), perhaps supported by inclusion of phospholipids in the affinity chromatography buffer. Tandem mass spectroscopy (MS/MS) of trypsin digested bands excised from the gel confirmed (with >95% confidence) that this band was due to alternative NADH dehydrogenase (NDH2) copurifying with complex I. The degree of NDH2 contamination in even the wild-type enzyme was highly variable among different preparations (compare the two wild-type samples in Fig. 2*B*) and was reflected in rates of NADH:DQ activity for the R121 variants, which showed a substantial piericidin-A-insensitive rate of NADH (rather than dNADH) oxidation (Fig. 2*C*).

As both NDUFS2-R121 variants exhibit matching subunit composition and functional effects, further functional and structural analyses focused on NDUFS2-R121M, owing to the higher amount of enzyme present in membranes and the correspondingly higher yields that were obtained from solubilization and purification. The catalytic activities of the purified wild-type and R121M complexes with dNADH (the complex-I-specific substrate) are compared in Figure 2*D*, confirming (as for the membrane-bound complexes in Fig. 1*C*) that the R121M variant lacks any significant quinone reductase activity. This result further confirms that the activity observed for the isolated enzyme in NADH:DQ assays is consequential of NDH2 contamination—not a piericidin-A-insensitive rate induced by structural changes around the N2 cluster and quinone-binding site.

Finally, to address the reason for the decreased content of the R121M complex in membranes, differential scanning fluorimetry (DSF) was used to compare the thermal stability of the R121M mutant and wild-type enzymes (Fig. S1). Surprisingly, there were no significant differences in the melting temperature (T_m_) between the DDM-soluble purified wild-type (T_m_ = 49.3 ± 0.2) and R121M (T_m_ = 48.9 ± 0.3) complexes or in the aggregation temperature (T_agg_ = 58.0 ± 0.4 for wild-type and 60.5 ± 0.9 for R121M). This further solidifies the notion that purified R121M complex I exists as an intact enzyme following purification; defects in enzyme assembly may explain the lower amount of R121 mutant complex I present in membranes.

### NDUFS2-R121 mutants show no N2 signal in EPR spectroscopy

**Figure 3:**
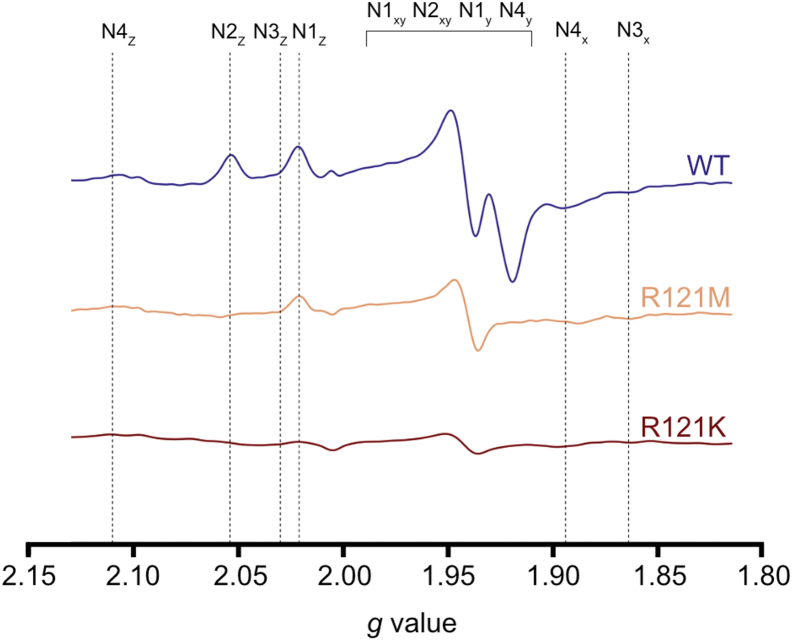
**EPR spectra of wild-type complex I and the R121 variants.** Purified samples were reduced with 2 mM sodium dithionite (final concentration) under anaerobic conditions. Measurements were performed at 15 K with 2 mW microwave power, 100 KHz modulation frequency and 7 G modulation amplitude. Spectra are not normalized for protein concentration (WT = 30 μM, R121M = 12 μM, R121K = 6 μM). The *g* values corresponding to individual FeS clusters in *Y. lipolytica* complex I are indicated (12).

Mutations in the second coordination sphere of the N2 cluster of complex I have been shown to evoke varied responses in the EPR signal for the reduced cluster, including shifts in the principal *g* values and decreased signal intensity, both indicative of a change in the cluster environment. Previous studies of the R121K mutant revealed no EPR signals attributable to complex I in membranes (30). However, as the low complex I content in membranes results in low signal to noise, it could not be determined if this was a specific effect on N2 or a global effect on the entire enzyme. Here, we used the purified, fully assembled enzyme to investigate the effects of the mutation on the EPR signals of the FeS clusters, particularly N2. The sodium dithionite (E_m7_ < −0.45 V) reduced spectra for the wild-type, R121M and R121K complexes recorded at 15 K and 2 mW are shown in Figure 3. The wild-type spectrum shows the four subspectra expected from four reduced FeS clusters in *Y. lipolytica* complex I under these measuring conditions (Fig. S2) (17). In comparison, the R121M and R121K mutants show a loss of signal at *g* = 2.05 and *g* = 1.92, which correspond to the characteristic *g*_z_ and *g*_x,y_ transitions of the N2 cluster, respectively. The loss of N2 signal intensity is in stark contrast to the other EPR detectable FeS clusters (N1b, N3, and N4) that provide clear contributions to the spectra of both mutants. Together with the results described above (Figs. 1 and 2), the spectra suggest the presence of a largely intact complex that either lacks the N2 cluster completely or contains a cluster with a reduction potential decreased substantially, to a value lower than that of the reductant used (note that we did not detect any EPR signals to suggest the reduced cluster switches from *S* = 1/2 to a higher spin state, see Fig. S2). Both effects would explain the impaired quinone reductase activity. EPR spectra of the oxidized R121 mutants did not exhibit any EPR signals (Fig. S2), thus the partial collapse of the [4Fe–4S] N2 cluster into a [3Fe–4S] cluster, which would be paramagnetic in its oxidized state, is not supported. As in the functional experiments above, the R121M mutant was superior to the R121K mutant in EPR analysis, further justifying it as the choice for structure determination.

### Structure of NDUFS2-R121M shows the wild-type-like N2 cluster is absent

**Figure 4:**
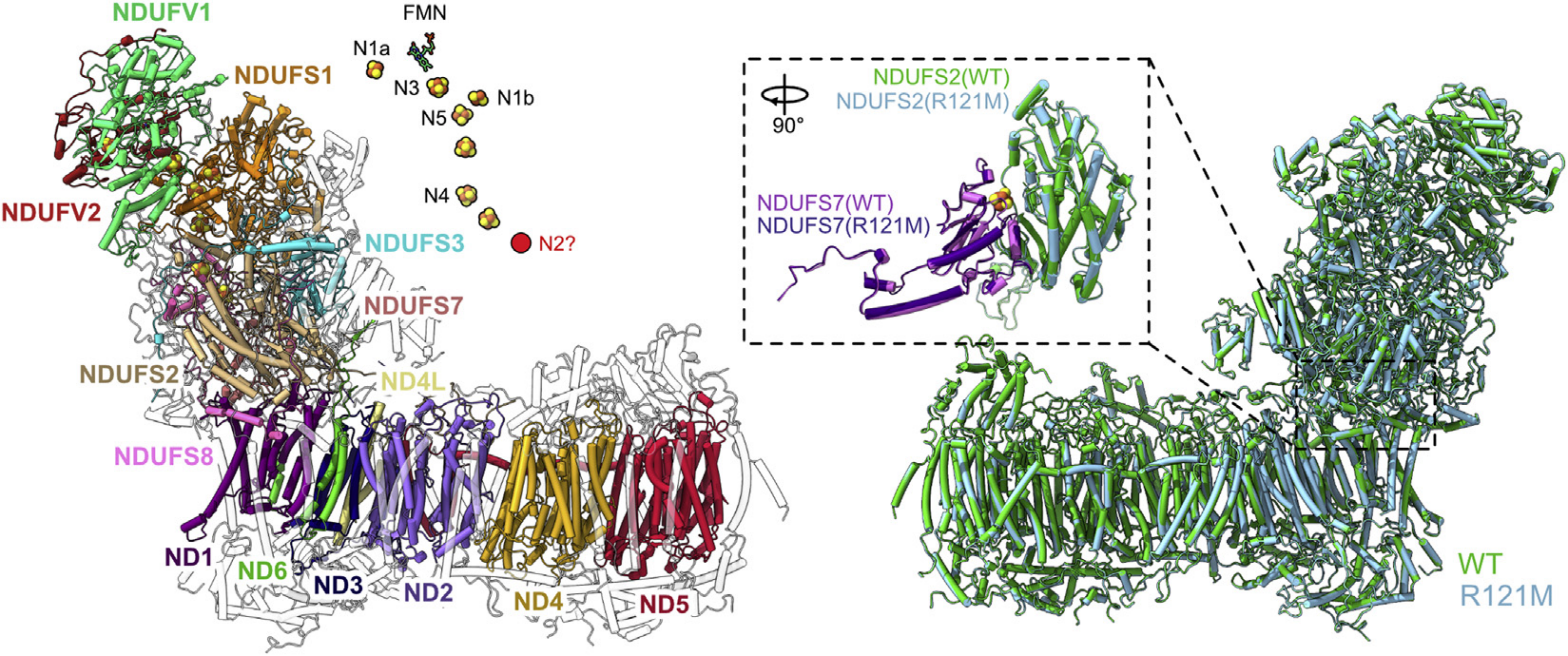
**Structure of the R121M variant of complex I from *Y. lipolytica*.** Overview of the structure of the R121M complex (3.7 Å resolution) shown with the 14 core (colored and labeled using *Y. lipolytica* nomenclature) and 28 supernumerary subunits (*white*) of *Y. lipolytica* complex I depicted as cartoons (the substoichiometric ST1 subunit is not present in this model). The *inset* shows the cofactor chain in a *stick*/*sphere representation*. The position where N2 is expected (but not modeled) is indicated. The alignment of the structures of wild-type (*green*, PDB ID: 6YJ4) and R121M (*light blue*) complex I (RMSD: 0.31 Å) is given in the right-hand figure. Models are depicted as *cartoons* to show the close match of secondary structural elements. The *inset* shows the N2 associated subunits NDUFS2 (wild-type = *green*, R121M = *light blue*) and NDUFS7 (wild-type = *light purple*, R121M = *indigo*) at a 90° rotation relative to the whole complex I. The N2 cluster of the wild-type model is shown for reference.

To probe the effect of the R121M mutation on complex I activity and on the N2 cluster, its structure was determined using cryo-EM. Purified R121M complex I (dNADH:DQ activity = 0.5 ± 0.1 μmol min^−1^ mg^−1^) at 2.8 mg ml^−1^ was frozen onto PEGylated gold grids and imaged using an FEI Titan Krios microscope with a Gatan K2 detector calibrated with a 1.07 Å pixel size. The data set of 2241 micrographs (66,568 particles) was processed in RELION 3.0 and 3.1 (45, 46) to a global map resolution of 3.7 Å (Figs. S3 and S4). The model of the 2.7 Å structure for the wild-type *Y. lipolytica* complex I (21) was fitted into the 3.7 Å map with UCSF Chimera (47). Dimethyl-R121 was mutated to methionine *in silico* in COOT (48) prior to real-space refinement of the model using PHENIX (49). All 14 core and 29 supernumerary subunits were present in the R121M structure (Fig. 4, Table S2), although the substoichiometric ST1 (18) was not modeled due to its poor resolution. The wild-type and R121M models were similar (with an overall RMSD of 0.31 Å), clearly signifying that global structural changes are not induced by the R121M mutation (Fig. 4, *right*). None of the core subunits showed any substantial changes in conformation and the NDUFS7 and NDUFS2 subunits, which surround the N2 cluster, displayed near-exact superimposition of their backbone polypeptide densities (Fig. 4). The structural analyses clearly demonstrate that the R121M mutant is an intact, wild-type like enzyme and that the effects of the mutation are strictly localized, with no long-range compositional or conformational changes.

**Figure 5:**
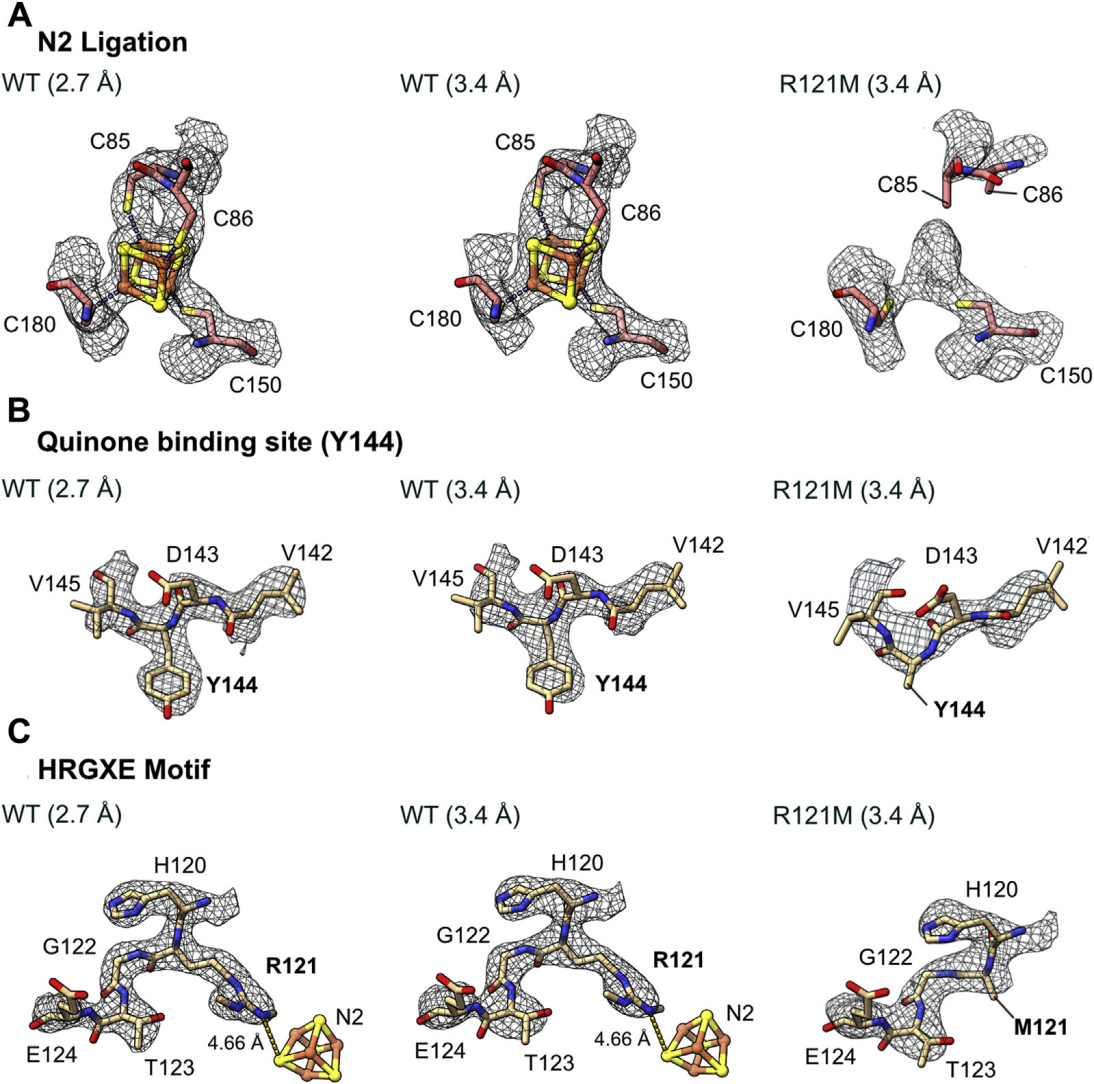
**Comparison of the N2 cluster coordination geometry and key regions in the wild-type and R121M complexes.***A*, the model and density map for N2 ligating residues shown for WT at optimum resolution (threshold = 0.04), WT filtered to 3.4 Å (threshold = 0.04), and R121M (threshold = 0.08). C85 and C86 are modeled as stubs due to the low confidence in the density map for these residues. *B*, models and density maps for the stretch of residues in NDUFS7 containing the Y144 ubiquinone ligating residue. Maps are shown for optimum resolution (threshold = 0.03), WT filtered to 3.4 Å (threshold = 0.03), and R121M (threshold = 0.08). Y144 residue modeled as a stub as a result of the absence of density. *C*, comparison of models and density maps for the HRGXE motif in the wild-type and R121M complexes. Map thresholds are the same as *B*. M121 is modeled as a stub due to the lack of density for the side chain in the map. Thresholds for densities were matched between WT and mutant maps by visual comparison.

**Figure 6:**
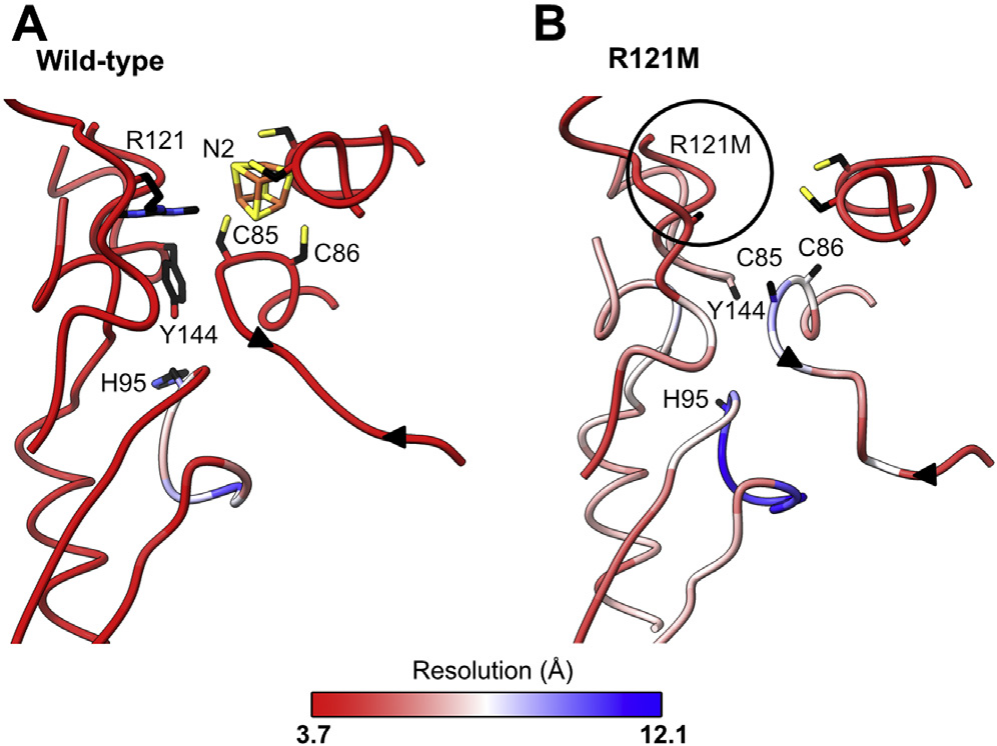
**The disorder induced at the N2 site in the R121M mutant compared with wild-type complex I.** Local resolution of the quinone binding site in wild-type complex I (filtered to 3.4 Å) (*A*) and R121M (*B*) complex I. The high degree of flexibility of the R121M side chain results in it not being modeled (*black circle*). This has knock-on effects on other residues. The tandem Cys residues that coordinate the N2 cluster in the WT are both mobile in the R121M complex I (no side chains modeled in *B*), likely due to no cluster coordination, and the loop that they are positioned on is no longer taut (indicated by *black arrowheads*). The cartoon backbone is colored according to local resolution on the scale shown. As expected from the wild-type structure (21), the NDUFS2 loop that contains the catalytic H95 is disordered, but in the R121M variant there is also disorder at the second catalytic ligand Y144 (no side chain modeled). Residues shown are identical for both structures (NDUFS2:139–150:448–466:84–103:115–125; NDUFS7:78–89:148–152:178–182).

The local resolution estimation (Fig. S4) at the N2 cluster position was approximately 3.4 Å, which is a sufficiently high to define the structural changes around the N2 cluster that result from the R121M mutation. Switching the dimethylated arginine to a methionine clearly perturbs the coordination of the cluster. The map of the wild-type complex contains a very clear map feature representing the cubane N2 cluster. This feature is replaced in the R121M map with a feature that is narrower and elongated, and that no longer supports the presence of a cubane FeS cluster (Fig. 5*A*). While this unknown cryo-EM density appears to retain the coordination of C150 and C180, no clear connectivity is observed from the tandem cysteine side chains (C85 and C86), which would complete the ligation of the N2 cluster. Only very weak map features suggest the possibility of transient and unstable interactions between C86 and the cluster-like density. Furthermore, the loop carrying the two liberated cysteine residues displays lower side chain definition than both its surroundings and the corresponding wild-type density, suggestive of its increased localized mobility. Overall, this whole region of the enzyme is notably less well resolved in comparison to in the wild-type map filtered to the same 3.4 Å resolution (Fig. 6). It is possible that R121 helps to stabilize the unfavorable geometry of the tandem cysteines, enabling the complete ligation and subsequent stability of the N2 cluster. This proposal is supported by the relaxation of the considerably strained backbone architecture in the wild-type to a more helical conformation in the mutant, in the absence of N2 coordination by the tandem cysteines (Fig. 6).

The R121 mutation propagates destabilization through the NDUFS2 and NDUFS7 subunits, causing further localized disorder in the quinone-binding channel (Fig. 6). The increased mobility afforded by the disconnected cysteines has a profound impact on nearby residues in NDUFS2. In contrast to in the wild-type enzyme, the side chain of NDUFS2-Y144, a quinone headgroup-ligating and N2-neighboring residue, lacks any clear density, indicating considerable flexibility in the variant compared with wild-type (Figs. 5*B* and 6). Y144 is in close proximity to C85 (the most mobile of the tandem cysteines) and A84, and the proximity of R121 to these residues suggests it plays a role in stabilizing the local structure. Though there is no direct interaction between R121 and the ligating cysteines, the dimethylated guanidium group extends to within ∼3.9 Å of A84, projecting a stabilizing influence on all the residues around the FeS cluster in the wild-type enzyme, which is lost when R121 is mutated to methionine. We also note increased mobility in the β1–β2 loop of NDUFS2, known to harbor residues essential to quinone binding and catalysis (27), with only the backbone of residues 90 to 96 of the catalytic β1–β2 loop clearly visible (Fig. 6). While both the wild-type and R121M maps show the complex in a mild deactive state (21), with the β1–β2 loop of NDUFS2 displaying some degree of dynamism, the increased flexibility in the R121M mutant indicates additional instability. The major secondary structural elements flanking this loop are well defined and consistent in both enzymes; however, the ND1 TMH5–6 and ND3 TMH1–2 loops that both contribute to the packing of this region also lack definition. Ultimately, the increased dynamics of catalytic elements important to quinone reduction indicate a role for the dimethylated R121 in ordering the quinone-binding site.

The highly conserved HRGXE motif in NDUFS2, which includes R121, was well ordered in both the wild-type and R121M complexes. All the side chains except that of the variant methionine were well resolved in the R121M enzyme (Fig. 5*C*), whereas the lack of a clear density for the methionine side chain again suggests a dynamic nature. This is in stark contrast to the very apparent cryo-EM density for dimethylated R121 in the wild-type enzyme. The flexibility of the methionine side chain is likely due to it sampling the large void created by removal of the bulky dimethylated arginine. Removing both the large side chain and the positive charge of the η-nitrogen of the arginine guanidinium group (see below) disrupts interactions with surrounding residues and destabilizes the N2 site, affecting the ligation of the FeS cluster and rendering the enzyme inactive for quinone reductase activity. Although the cryo-EM map around the N2 site of the R121M mutant is not high enough resolution to assign the feature at the N2 position, the (partial) loss of the coordinated FeS cluster and the subsequent disordering of the quinone-binding site explain the lack of catalytic activity in R121M complex I.

## Discussion

Understanding the mechanisms by which complex I mutations affect quinone reductase activity is a vital step in determining the roles of individual residues in structure and function. Here, we focused on the widely conserved NDUFS2-R121 residue, located in close proximity to the N2 cluster from where it may influence its magnetic and redox properties. Furthermore, R121 is of particular interest because its dimethylation, in many species of complex I, appears to be important for enzyme assembly or maturation (35, 36) and because there are striking similarities in the biochemical and spectroscopic properties of variants of NDUFS2-R121 and NDUFS2-R141. While both residues are part of the secondary coordination sphere of N2, R141 has been studied in much greater detail (25, 33). Here we have shown, using single-particle cryo-EM and EPR spectroscopy, that dimethyl-R121 is essential for the assembly and/or stability of both the N2 FeS cluster and the adjacent quinone-binding site in complex I, with its mutations causing loss of quinone reductase activity within an otherwise native-like structure.

Work on the NDUFS2-R141(M/K) mutants in *Y. lipolytica* (25) first led to the idea that complex I could assemble and reduce ubiquinone without a functional N2 cluster. This controversial interpretation was challenged with the alternative suggestion that (provided the catalytic activity is retained) the absence of the N2 EPR signal is more likely to stem from a decrease in its reduction potential (50). Similar suggestions have been made for mutants of R121, but in our case, loss of the N2 EPR signal results from the absence of a well-ordered, stable N2 cluster, not just from a change in its properties. However, it is difficult to confidently extend our current results to the previous studies because both R121 mutants investigated here are devoid of quinone reductase activity (in both membrane-bound and detergent-solubilized form), whereas the previous studies on both R121 (27, 30) and R141 (25) variants described some retention of activity. Note that we chose not to normalize our membrane activities to their complex I contents (determined using the dNADH:APAD^+^ assay) as this approach amplifies the low background activities and that the consistently high levels of NDH2 contamination we observed may further give misleading results. Work with the R274A variant of *Escherichia coli* complex I (homologous to *Y. lipolytica* R141) described loss of both activity and EPR in membranes (51), although higher activity in the NADH:DQ assay (22% of wild-type) with the purified enzyme and proteoliposomes. Although direct comparisons are complicated by the atypical EPR characteristics of *E. coli* N2 and differences in the cluster environments in the two species (43), it is also possible that mutations in R121 and R141 induce similar effects by different mechanisms. We conclude that mutations of R121M/K lead to loss of a functional N2 cluster, precluding quinone reduction, and also to loss of structural integrity in the quinone-binding site, preventing or hindering quinone binding. There is no true N2-independent method of quinone reduction, and conclusions about the effects of the positive charge or methylation of R121 on the N2 cluster cannot be drawn. Our results highlight the power of structural analyses in interpreting the functional effects of site-directed mutants, and the perils of overinterpreting functional data in a specific way, without clear knowledge of the extent and range of effects (both specific and nonspecific) that the mutations have caused.

While our structural data imply the absence of a canonical N2 cluster in the R121M variant, the feature that replaces it is difficult to identify. The cryo-EM density is more elongated than in wild-type, and it is not a well-ordered, strong density like the densities of the other [4Fe-4S] clusters present. First, the cubane N2 cluster may remain intact, but be “suspended” in the protein by just two cysteine ligands and so conformationally mobile. We know of no precedence for such a partially coordinated [4Fe-4S] cluster, but expect that such a ligand change would have a substantial impact on the reduction potential (52). Furthermore, our undefined density resembles that observed previously for the partially disconnected cluster observed in the NADH/dithionite-reduced hydrophilic arm of *T. thermophilus* complex I (53). As in our R121M mutant, EPR spectroscopy on this damaged hydrophilic domain, formed by cleavage of the complex along a fracture line adjacent to the cluster, does not exhibit the expected signature of N2 (41, 43). However, the signature of a canonical N2 cluster has not been observed in *T. thermophilus* membrane particles either (42), rendering this observation inconclusive. Second, the unknown density may indicate replacement of the FeS cluster with a different cofactor, such as a mononuclear metal centre. EPR spectra of the oxidized enzymes did not show signals indicative of the presence of Cu(II), Co(II), or Fe(III), and no evidence could be obtained for high-spin Fe(II), although detecting this *S* = 2 species would likely require parallel-mode measurements and higher concentrations than we were able to achieve. The identity of the species present in the N2 site thus remains unknown.

**Figure 7:**
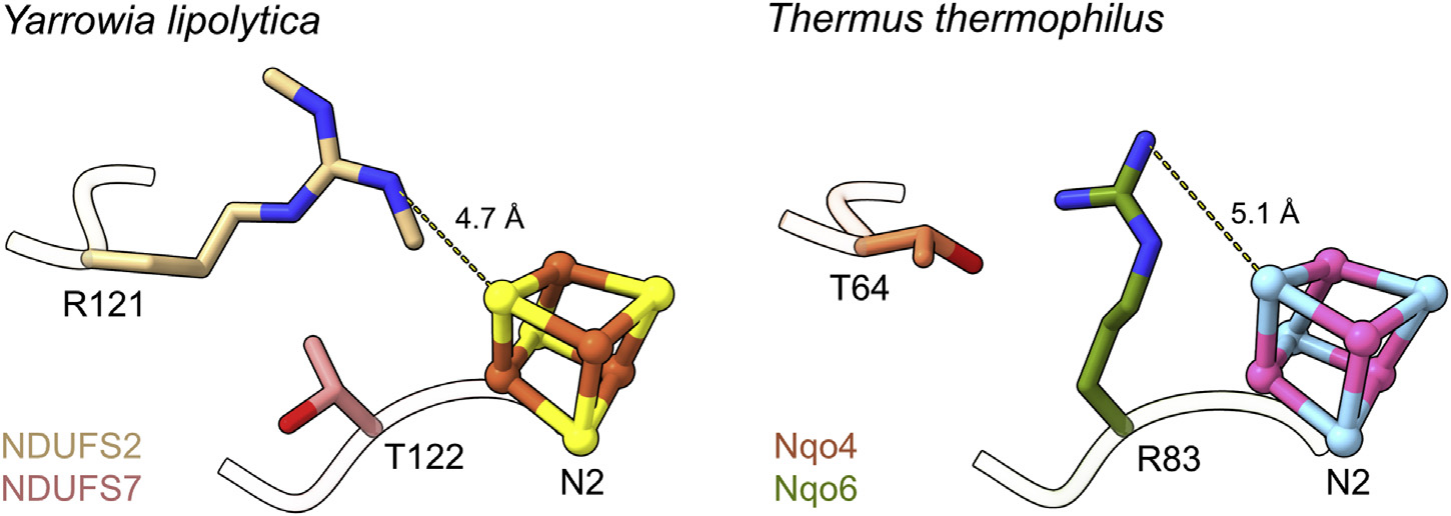
**The intersubunit residue switch between *Y. lipolytica* and *T. thermophilus*.** Structures of complex I from *Y. lipolytica* (PDB ID: 6YJ4) and *T. thermophilus* (PDB ID: 4HEA) displaying the *Y. lipolytica* subunits NDUFS2 (*wheat*) and NDUFS7 (*pink*) and homologous *T. thermophilus* subunits Nqo4 (*orange*) and Nqo6 (*green*). The N2 cluster is shown in *brown*/*yellow* for *Y. lipolytica* and *pink*/*blue* for *T. thermophilus*. The side chains from NDUFS2-R121 and NDUFS7-R83 occupy the same space. Distances are shown between the two closest guanidium group nitrogens and FeS sulfides.

In complex I from *T. thermophilus*, the equivalent arginine to NDUFS2-R121 is substituted by a threonine, which appears to undermine its critical role in stabilizing N2 (41, 42). However, further investigation revealed an intersubunit residue switch: in a further reverse change, an arginine (R83) in *T. thermophilus* NDUFS7 (Nqo6) occupies the position of a threonine, NDUFS7-T122, in *Y. lipolytica* (54), and comparison of the structures from *T. thermophilus* and *Y. lipolytica* shows that the two arginine side chains overlap the same space (Fig. 7). The two arginines thus likely share the same functional and structural role. Simulations have suggested that *T. thermophilus* R83 plays a vital role in complex I function by electrostatically stabilizing the anionic Y87 (NDUFS2-Y144 in *Y. lipolytica*) following its deprotonation by ubiquinone (54), and we note a noticeable loss of density for Y144 in our mutant. Additional simulations on *T. thermophilus* complex I have further implicated the arginine as a redox-switch that moves in response to the redox state of N2 to control quinone access (55). *In silico* mutations have also suggested its importance for the structural stability of subunit interfaces (55). Unfortunately, loss of a functional N2 cluster in our R121 mutants precludes deconvolution of these different compound effects.

Finally, the possible role that R121 dimethylation plays in assembly is intriguing. A comprehensive analysis of the dimethylation of NDUFS2-R85 (R121) in human 143B osteosarcoma cells showed a clear correlation between the level of dimethylation and mature complex I assembly in siRNA knockdowns of the methyltransferase responsible for the posttranslational modification (36). Furthermore, when methylation was impaired, decreased levels of ND1 and NDUFS7 were concomitant with loss of a NDUFS2-containing 400 kDa assembly intermediate and accumulation of a NDUFB8-containing ∼460 kDa subcomplex (36). Consistent effects are observed when methylation of NDUFS2 is perturbed in zebrafish (56) and the amoeba *Dictyostelium discoideum* (37, 57). Our R121M/K complexes I support these observations in part, with substantial (60–75%) decreases in complex I membrane enrichment. We suggest that the “intersubunit” arginine acts as part of a key/lock mechanism, helping to hold NDUFS2 and NDUFS7 together at a crucial point in the assembly process (Fig. 7). In *T. thermophilus*, the arginine being present in the same subunit as the cluster may reflect different challenges to assembly in thermophilic organisms. Without a functional key/lock, a substantial proportion of the nascent enzyme fails to mature in *Yarrowia*, while a smaller proportion continues the assembly process to give a purifiable, intact protein with an overall wild-type structure. We note that dimethylation of R121 has been demonstrated in a diverse array of complex I homologues, but the corresponding arginine in *E. coli* complex I is not dimethylated (35), and this may contribute to the lower reduction potential (*ca.* −220 mV) of the N2 cluster in *E. coli* than in other species (typically −150 mV) (43, 58). Here, the R121K mutation was chosen to preserve the positive charge of the dimethylated arginine (59) and the R121M mutation to space fill and confer hydrophobicity in the absence of charge, and both mutants yielded similar biochemical and spectroscopic results. In the future, suppressing the *Y. lipolytica* analogue to the NDUFAF7 methylase may allow the specific roles of the posttranslational modification in assembly and catalysis to be determined independently of the presence of the arginine residue itself.

## Experimental procedures

### Plasmid preparation and transformation

The pUB26-NUCM plasmid was provided by Prof. U. Brandt (Radboud Institute for Molecular Life Sciences). Site-directed mutagenesis was carried out by PCR using Q5 polymerase (New England Biolabs) with nonoverlapping primers. The linear products were 5′-phosphorylated, blunt-end-ligated and transformed into *E. coli* DH5-Alpha competent cells (New England Biolabs) for sequencing. Plasmid transformations were performed as described previously (60). In brief, Δnucm cells (provided by Prof. U. Brandt) were grown at 30 °C in 2xYPD overnight, collected and kept on ice before being washed three times in ice-cold buffer containing 10 mM Tris-HCl (pH 7.5), 0.6 M sorbitol, 150 mM Li-acetate. Approximately 1 μg of plasmid DNA was added to 200 μl of cells and electroporated with a single pulse at 1.5 kV, 200 Ω, and 25 μF (pulse length ∼4.6 ms). Cells were incubated at 30 °C for 2 to 3 h, then plated onto YPD containing 150 μg ml^−1^ hygromycin B, and incubated for 3 days. Single transformant colonies were selected, confirmed by sequencing, and grown in 2xYPD with hygromycin B. Cell stocks were frozen in 50% (v/v) glycerol.

### Preparation of mitochondrial membranes from *Y. lipolytica*

*Y. lipolytica* cells of wild-type (GB10 from Prof. U. Brandt (16)), R121M (GB10 Δ*nucm* + pUB26 *nucm*–R121M) and R121K (GB10 Δ*nucm* + pUB26 *nucm*–R121K) were grown on 2xYPD media and harvested as described previously (44). Mitochondrial membranes were prepared as follows (44, 61): ∼300 g of cells were resuspended into breaking buffer (20 mM MOPS (pH 7.2), 400 mM sorbitol, 5 mM EDTA, 2 mM benzamidine chloride, 2% (w/v) bovine serum albumin (BSA), and 5 mM ε-aminocaproic acid) to a volume of ∼600 ml, then 800 μM phenylmethylsulfonyl fluoride (PMSF) added. The resulting suspension was lysed by two passages through a DYNO-MILL (Willy A. Bachofen UK). Unbroken cells and debris were removed by centrifugation (5500*g*, 15 min, 4 °C), and then the membranes were collected (208,000*g*, 1 h, 4 °C) and resuspended in washing buffer (20 mM MOPS (pH 7.2), 400 mM sorbitol, 2 mM benzamidine chloride, 5 mM ε-aminocaproic acid). The membranes were collected by centrifugation and resuspended two more times, with the time of centrifugation reduced to 45 min and 35 min for the second and third washes, respectively, then finally suspended in 20 mM NaH_2_PO_4_ (pH 7.4), 150 mM NaCl, 10% (v/v) glycerol to 20 to 30 mg ml^−1^ before freezing in liquid N_2_ and storage at −80 °C. Total protein concentration in the membrane was determined by bicinchoninic acid (BCA) assay.

### Purification of complex I from mitochondrial membranes

Complex I was purified as described previously (16, 44) with some adaptations for preparation of cryo-EM grids (21). All purification steps were performed at 4 °C. Approximately 200 mg membrane protein containing complex I was solubilized at a 2:1 weight ratio with *n*-dodecyl-β-D-maltoside (DDM, Glycon) for 30 min following the addition of the cOmplete, EDTA-free protease inhibitor cocktail (Roche). Solubilized material was separated by centrifugation at 180,000*g* for 45 min, then NaCl and neutralized imidazole were added to 400 mM and 20 mM, respectively. The sample was passed through a 0.22 μm syringe filter, then loaded onto a 5 ml HisTrap HP column (GE Life Sciences) pre-equilibrated with 20 mM NaH_2_PO_4_ (pH 7.4), 400 mM NaCl, 52 mM imidazole, 0.2% (w/v) DDM, 0.02% (w/v) asolectin (Avanti) and 0.02% (w/v) CHAPS (Calbiochem). The column was washed with the same buffer, then complex I was eluted with 140 mM imidazole. Complex-I-containing fractions were pooled, concentrated to ∼100 μl using an Amicon Ultra (100 MWCO) 0.5 ml centrifugal concentrator (Merck-Milipore), and applied to a Superose 6 Increase 5/150 gel filtration column (GE Life Sciencecs) pre-equilibrated with 20 mM MOPS (pH 7.45), 150 mM NaCl, 0.05% (w/v) DDM. The highest concentration fractions (as determined by NanoDrop, Thermo Scientific) were used for cryo-EM grid preparation. The concentrations of samples used for compositional and functional assays were determined using the BCA assay.

### Functional characterization of complex I

All activity assay measurements were recorded using a Spectramax Plus 384 plate reader (Molecular Devices). Membrane activity ((d)NADH:O_2_) measurements of complexes I–III–IV were performed at 32 °C in 20 mM Tris-HCl (pH 7.5 at 32 °C), 250 mM sucrose with 25 μg ml^−1^ membranes, 5 μM cytochrome *c* (from *Saccharomyces cerevisiae*, Sigma-Aldrich), and 200 μM (d)NADH (Sigma-Aldrich). Piericidin A (Santa Cruz Biotechnology) was added at a final concentration of 1 μM to inhibit complex I when required. NADH oxidation was monitored at 340 to 380 nm (ε = 4.81 mM^−1^ cm^−1^). The activity of the purified enzyme ((d)NADH:decylubiquinone, DQ) was measured similarly, in the same buffer with 0.5 μg ml^−1^ protein, 200 μM DQ (Sigma-Aldrich), 0.15% (w/v) asolectin, 0.15% (w/v) CHAPS, and 200 μM (d)NADH. dNADH:APAD^+^ activity was measured in 10 mM Tris-SO_4_ (pH 7.5), 50 mM KCl with 20 μg ml^−1^ membranes, 500 μM APAD^+^ (Sigma-Aldrich), 15 μg ml^−1^ alamethicin (Stratech), 500 nm piericidin A, and 100 μM dNADH and monitored at 400 to 450 nm (ε = 3.16 mM^−1^ cm^−1^).

### Composition of membranes and soluble complex I

BN-PAGE was performed as described previously (44). Membranes (6 mg ml^−1^) were treated with DDM at a 2:1 weight ratio, agitated for 30 min at 4 °C, and then centrifuged (160,000*g*, 30 min, 4 °C). In total, 40 μg of protein was applied to each lane of a NativePAGE 3 to 12% Bis-Tris gel (Invitrogen) and the gel run according to the manufacturer’s instructions. Proteins were visualized by Coomassie R-250 staining or by the in-gel NADH oxidase activity assay, using 20 mM Tris-HCl (pH 7.0) supplemented with 150 mM NADH and 0.5 mg ml^−1^ nitrotetrazolium blue (NBT, Sigma-Aldrich). NBT is a nonspecific oxidizing agent that produces a blue product when reduced by a reduced flavin, such as that formed by NADH in complex I (62). BN-PAGE of purified complex I was performed similarly, except without the solubilization step and using 20 μg of total protein. SDS-PAGE of purified complex I was performed using a Novex WedgeWell 10 to 20% Tris-glycine gel according to the manufacturer’s instructions.

### Thermostability assays of purified complex I

Complex I (1 mg/ml) was loaded into a capillary tube and analyzed in a NanoTemper Prometheus NT.48 differential scanning fluorimeter (DSF). The temperature was increased from 15 to 95 °C (3 °C min^−1^) and protein unfolding monitored *via* the tryptophan fluorescence (350/330 nm). Protein aggregation was measured concurrently as the light scattering during denaturation. Melting temperature (T_m_) and aggregation temperature (T_agg_) were determined as the temperature at which the protein was 50% unfolded or 50% aggregated, respectively.

### EPR spectroscopy

EPR samples of complex I were prepared by reduction with 2 mM sodium dithionite (final concentration) under anaerobic conditions in a Braun UniLab plus glovebox at room temperature (O_2_ < 0.5 ppm, N_2_ atmosphere). Sample concentrations were determined before EPR sample preparation using the BCA assay. Samples were measured at X-band (9.5 GHz) using a X/Q-band Bruker Elexsys E580 spectrometer (Bruker BioSpin GmbH) equipped with a closed-cycle cryostat (Cryogenic Ltd) and using a X-band split-ring resonator (ER 4118X-MS2) as described previously (63). Specific measurement conditions are given in figure captions. EPR simulations were performed using the EasySpin package for MATLAB (64). Signals were quantified relative to each other on the basis of the intensities of their simulated spectra, determined by integration.

### Cryo-EM grid preparation and data collection

UltrAuFoil 0.6/1 gold grids (Quantifoil) were glow discharged at 20 mA for 60 s and PEGylated by anaerobic incubation in a solution of 5 mM 11-mercaptoundecyl hexaethyleneglycol in ethanol for 5 days (65). Grids were washed with ethanol and air-dried before use. Grids were prepared using an FEI Vitrobot Mark IV set to 100% relative humidity at 4 °C. In total, 2.5 μl complex I from the peak chromatography fractions (2.8 mg ml^−1^) was applied to the grids and blotted for 10 s with a blot force of −10 or −9 before plunge freezing in liquid ethane. Data collection (Department of Biochemistry, University of Cambridge) was performed using an FEI Titan Krios with a K2 counting mode detector resulting in 2241 micrograph images (21). Collection parameters are given in Table S1. A total dose of 47.9 electrons/Å^2^ was used over 40 frames with an exposure time of 10 s.

### Cryo-EM data processing

Data were processed using the RELION-3.1 (45, 66) pipeline shown in Fig. S3. Briefly, beam-induced motion correction was performed with MotionCor2 (67) followed by CTF estimation using GCTF (68). In total, 369 micrographs containing bad ice were removed. Automated particle picking using a 3D reference yielded 33,281 particles, which were used as an initial 3D auto-refinement before CTF refinement and Bayesian polishing (66) to provide better-quality particles for subsequent 3D classification over five classes. Three classes (∼21K particles) were combined for additional CTF refinement (including higher-order corrections (46)), Bayesian polishing, and 3D auto-refinement before a final 3.7 Å map was generated from 21,013 particles. The output map had a pixel size of 1.05, downsized to match the wild-type map, differing from the 1.07 calibrated pixel size. Output maps were subjected to local sharpening using MonoRes (69) and LocalDeblur (70) in the Scipion (71) package.

### Model building

The model was built (Table S2) starting by rigid-body fitting the near-complete wild-type 2.7 Å model (PDB: 6YJ4) (21) into the 3.7 Å map obtained here using UCSF Chimera (47). The protein was locally inspected and remodeled (including side chain clipping), and lipids and detergent molecules updated to match the signal in the 3.7 Å map using Coot (48). Multiple rounds of PHENIX real-space refinement (49) were performed with local remodeling, with close attention paid to the site of the R121M. Model validation and overfitting analysis (72, 73) were performed (Table S1 and Fig. S4).

## Data availability

Data accession codes: EMD-11969, PDB ID: 7B0N. Other data supporting the findings of this manuscript are available from the corresponding authors upon reasonable request.

## Supporting information

This article contains supporting information (20, 45, 46, 72, 73, 74, 75).

## Conflict of interest

The authors declare that they have no conflicts of interest with the contents of this article.

## References

[1] Hirst J. (2013). Mitochondrial complex I. Annu. Rev. Biochem..

[2] Parey K., Wirth C., Vonck J., Zickermann V. (2020). Respiratory complex I — structure, mechanism and evolution. Curr. Opin. Struct. Biol..

[3] Kaila V.R.I. (2018). Long-range proton-coupled electron transfer in biological energy conversion: Towards mechanistic understanding of respiratory complex I. J. R. Soc. Interface.

[4] Fassone E., Rahman S. (2012). Complex I deficiency: Clinical features, biochemistry and molecular genetics. J. Med. Genet..

[5] Murphy M.P. (2009). How mitochondria produce reactive oxygen species. Biochem. J..

[6] Agip A.-N.A., Blaza J.N., Fedor J.G., Hirst J. (2019). Mammalian respiratory complex I through the lens of cryo-EM. Annu. Rev. Biophys..

[7] Galemou Yoga E., Angerer H., Parey K., Zickermann V. (2020). Respiratory complex I – mechanistic insights and advances in structure determination. Biochim. Biophys. Acta.

[8] Agip A.N.A., Blaza J.N., Bridges H.R., Viscomi C., Rawson S., Muench S.P., Hirst J. (2018). Cryo-EM structures of complex I from mouse heart mitochondria in two biochemically defined states. Nat. Struct. Mol. Biol..

[9] Letts J.A., Fiedorczuk K., Degliesposti G., Skehel M., Sazanov L.A. (2019). Structures of respiratory supercomplex I+III_2_ reveal functional and conformational crosstalk. Mol. Cell.

[10] Parey K., Brandt U., Xie H., Mills D.J., Siegmund K., Vonck J., Kühlbrandt W., Zickermann V. (2018). Cryo-EM structure of respiratory complex I at work. Elife.

[11] Kampjut D., Sazanov L.A. (2020). The coupling mechanism of mammalian respiratory complex I. Science.

[12] Kerscher S., Dröse S., Zwicker K., Zickermann V., Brandt U. (2002). *Yarrowia lipolytica*, a yeast genetic system to study mitochondrial complex I. Biochim. Biophys. Acta.

[13] Tocilescu M.A., Fendel U., Zwicker K., Kerscher S., Brandt U. (2007). Exploring the ubiquinone binding cavity of respiratory complex I. J. Biol. Chem..

[14] Cabrera-Orefice A., Yoga E.G., Wirth C., Siegmund K., Zwicker K., Guerrero-Castillo S., Zickermann V., Hunte C., Brandt U. (2018). Locking loop movement in the ubiquinone pocket of complex I disengages the proton pumps. Nat. Commun..

[15] Galemou Yoga E., Haapanen O., Wittig I., Siegmund K., Sharma V., Zickermann V. (2019). Mutations in a conserved loop in the PSST subunit of respiratory complex I affect ubiquinone binding and dynamics. Biochim. Biophys. Acta.

[16] Kashani-Poor N., Kerscher S., Zickermann V., Brandt U. (2001). Efficient large scale purification of his-tagged proton translocating NADH:ubiquinone oxidoreductase (complex I) from the strictly aerobic yeast *Yarrowia lipolytica*. Biochim. Biophys. Acta.

[17] Djafarzadeh R., Kerscher S., Zwicker K., Radermacher M., Lindahl M., Schägger H., Brandt U. (2000). Biophysical and structural characterization of proton-translocating NADH-dehydrogenase (complex I) from the strictly aerobic yeast *Yarrowia lipolytica*. Biochim. Biophys. Acta.

[18] D’Imprima E., Mills D.J., Parey K., Brandt U., Kühlbrandt W., Zickermann V., Vonck J. (2016). Cryo-EM structure of respiratory complex I reveals a link to mitochondrial sulfur metabolism. Biochim. Biophys. Acta.

[19] Zickermann V., Wirth C., Nasiri H., Siegmund K., Schwalbe H., Hunte C., Brandt U. (2015). Mechanistic insight from the crystal structure of mitochondrial complex I. Science.

[20] Parey K., Haapanen O., Sharma V., Köfeler H., Züllig T., Prinz S., Siegmund K., Wittig I., Mills D.J., Vonck J., Kühlbrandt W., Zickermann V. (2019). High-resolution cryo-EM structures of respiratory complex I: Mechanism, assembly, and disease. Sci. Adv..

[21] Grba D.N., Hirst J. (2020). Mitochondrial complex I structure reveals ordered water molecules for catalysis and proton translocation. Nat. Struct. Mol. Biol..

[22] Sazanov L.A., Hinchliffe P. (2006). Structure of the hydrophilic domain of respiratory complex I from *Thermus thermophilus*. Science.

[23] Ingledew W.J., Ohnishi T. (1980). An analysis of some thermodynamic properties of iron-sulphur centres in site I of mitochondria. Biochem. J..

[24] Kashani-Poor N., Zwicker K., Kerscher S., Brandt U. (2001). A central functional role for the 49-kDa subunit within the catalytic core of mitochondrial complex I. J. Biol. Chem..

[25] Grgic L., Zwicker K., Kashani-Poor N., Kerscher S., Brandt U. (2004). Functional significance of conserved histidines and arginines in the 49-kDa subunit of mitochondrial complex I. J. Biol. Chem..

[26] Zwicker K., Galkin A., Dröse S., Grgic L., Kerscher S., Brandt U. (2006). The Redox-Bohr group associated with iron-sulfur cluster N2 of complex I. J. Biol. Chem..

[27] Tocilescu M.A., Zickermann V., Zwicker K., Brandt U. (2010). Quinone binding and reduction by respiratory complex I. Biochim. Biophys. Acta.

[28] Flemming D., Schlitt A., Spehr V., Bischof T., Friedrich T. (2003). Iron-sulfur cluster N2 of the *Escherichia coli* NADH:ubiquinone oxidoreductase (complex I) is located on subunit NuoB. J. Biol. Chem..

[29] Duarte M., Pópulo H., Videira A., Friedrich T., Schulte U. (2002). Disruption of iron-sulphur cluster N2 from NADH:ubiquinone oxidoreductase by site-directed mutagenesis. Biochem. J..

[30] Tocilescu M.A. (2009). The Ubiquinone and Inhibitor Binding Pocket of Complex I from Yarrowia lipolytica: A Structure-Based Mutagenesis Study.

[31] Fendel U., Tocilescu M.A., Kerscher S., Brandt U. (2008). Exploring the inhibitor binding pocket of respiratory complex I. Biochim. Biophys. Acta.

[32] Le Breton N., Wright J.J., Jones A.J.Y., Salvadori E., Bridges H.R., Hirst J., Roessler M.M. (2017). Using hyperfine electron paramagnetic resonance spectroscopy to define the proton-coupled electron transfer reaction at Fe–S cluster N2 in respiratory complex I. J. Am. Chem. Soc..

[33] Galkin A., Brandt U. (2005). Superoxide radical formation by pure complex I (NADH:ubiquinone oxidoreductase) from *Yarrowia lipolytica*. J. Biol. Chem..

[34] Albracht S.P.J. (1993). Intimate relationships of the large and the small subunits of all nickel hydrogenases with two nuclear-encoded subunits of mitochondrial NADH: Ubiquinone oxidoreductase. Biochim. Biophys. Acta.

[35] Carroll J., Ding S., Fearnley I.M., Walker J.E. (2013). Post-translational modifications near the quinone binding site of mammalian complex I. J. Biol. Chem..

[36] Rhein V.F., Carroll J., Ding S., Fearnley I.M., Walker J.E. (2013). NDUFAF7 methylates arginine 85 in the NDUFS2 subunit of human complex I. J. Biol. Chem..

[37] Carilla-Latorre S., Gallardo M.E., Annesley S.J., Calvo-Garrido J., Graña O., Accari S.L., Smith P.K., Valencia A., Garesse R., Fisher P.R., Escalante R. (2010). MidA is a putative methyltransferase that is required for mitochondrial complex I function. J. Cell Sci..

[38] Fiedorczuk K., Sazanov L.A. (2018). Mammalian mitochondrial complex I structure and disease-causing mutations. Trends Cell Biol..

[39] Tuppen H.A.L., Hogan V.E., He L., Blakely E.L., Worgan L., Al-Dosary M., Saretzki G., Alston C.L., Morris A.A., Clarke M., Jones S., Devlin A.M., Mansour S., Chrzanowska-Lightowlers Z.M.A., Thorburn D.R. (2010). The p.M292T NDUFS2 mutation causes complex I-deficient Leigh syndrome in multiple families. Brain.

[40] Baradaran R., Berrisford J.M., Minhas G.S., Sazanov L.A. (2013). Crystal structure of the entire respiratory complex I. Nature.

[41] Hinchliffe P., Carroll J., Sazanov L.A. (2006). Identification of a novel subunit of respiratory complex I from *Thermus thermophilus*. Biochemistry.

[42] Meinhardt S.W., Wang D.C., Hon-Nami K., Yagi T., Oshima T., Ohnishi T. (1990). Studies on the NADH-menaquinone oxidoreductase segment of the respiratory chain in *Thermus thermophilus* HB-8. J. Biol. Chem..

[43] Hirst J., Roessler M.M. (2016). Energy conversion, redox catalysis and generation of reactive oxygen species by respiratory complex I. Biochim. Biophys. Acta.

[44] Varghese F., Atcheson E., Bridges H.R., Hirst J. (2015). Characterization of clinically identified mutations in NDUFV1, the flavin-binding subunit of respiratory complex I, using a yeast model system. Hum. Mol. Genet..

[45] Zivanov J., Nakane T., Forsberg B.O., Kimanius D., Hagen W.J.H., Lindahl E., Scheres S.H.W. (2018). New tools for automated high-resolution cryo-EM structure determination in RELION-3. Elife.

[46] Zivanov J., Nakane T., Scheres S.H.W. (2020). Estimation of high-order aberrations and anisotropic magnification from cryo-EM data sets in RELION-3.1. IUCrJ.

[47] Pettersen E.F., Goddard T.D., Huang C.C., Couch G.S., Greenblatt D.M., Meng E.C., Ferrin T.E. (2004). UCSF Chimera - a visualization system for exploratory research and analysis. J. Comput. Chem..

[48] Emsley P., Lohkamp B., Scott W.G., Cowtan K. (2010). Features and development of Coot. Acta Crystallogr. D Biol. Crystallogr..

[49] Adams P.D., Afonine P.V., Bunkóczi G., Chen V.B., Davis I.W., Echols N., Headd J.J., Hung L.W., Kapral G.J., Grosse-Kunstleve R.W., McCoy A.J., Moriarty N.W., Oeffner R., Read R.J., Richardson D.C. (2010). Phenix: A comprehensive Python-based system for macromolecular structure solution. Acta Crystallogr. D Biol. Crystallogr..

[50] Vinogradov A.D., Grivennikova V.G. (2016). Oxidation of NADH and ROS production by respiratory complex I. Biochim. Biophys. Acta.

[51] Belevich G., Euro L., Wikström M., Verkhovskaya M. (2007). Role of the conserved arginine 274 and histidine 224 and 228 residues in the NuoCD subunit of complex I from *Escherichia coli*. Biochemistry.

[52] Bak D.W., Elliott S.J. (2014). Alternative FeS cluster ligands: Tuning redox potentials and chemistry. Curr. Opin. Chem. Biol..

[53] Berrisford J.M., Sazanov L.A. (2009). Structural basis for the mechanism of respiratory complex I. J. Biol. Chem..

[54] Haapanen O., Sharma V. (2017). Role of water and protein dynamics in proton pumping by respiratory complex I. Sci. Rep..

[55] Gupta C., Khaniya U., Chan C.K., Dehez F., Shekhar M., Gunner M.R., Sazanov L.A., Chipot C., Singharoy A. (2020). Charge transfer and chemo-mechanical coupling in respiratory complex I. J. Am. Chem. Soc..

[56] Zurita Rendón O., Silva Neiva L., Sasarman F., Shoubridge E.A. (2014). The arginine methyltransferase NDUFAF7 is essential for complex I assembly and early vertebrate embryogenesis. Hum. Mol. Genet..

[57] Shahul Hameed U.F., Sanislav O., Lay S.T., Annesley S.J., Jobichen C., Fisher P.R., Swaminathan K., Arold S.T. (2018). Proteobacterial origin of protein arginine methylation and regulation of complex I assembly by MidA. Cell Rep..

[58] Leif H., Sled V.D., Ohnishi T., Weiss H., Friedrich T. (1995). Isolation and characterization of the proton-translocating NADH:ubiquinone oxidoreductase from *Escherichia coli*. Eur. J. Biochem..

[59] Evich M., Stroeva E., Zheng Y.G., Germann M.W. (2016). Effect of methylation on the side-chain pKa value of arginine. Protein Sci..

[60] Wang J.-H., Hung W., Tsai S.-H. (2011). High efficiency transformation by electroporation of *Yarrowia lipolytica*. J. Microbiol..

[61] Birrell J.A.A., Morina K., Bridges H.R.R., Friedrich T., Hirst J. (2013). Investigating the function of [2Fe–2S] cluster N1a, the off-pathway cluster in complex I, by manipulating its reduction potential. Biochem. J..

[62] Wittig I., Karas M., Schägger H. (2007). High resolution clear native electrophoresis for in-gel functional assays and fluorescence studies of membrane protein complexes. Mol. Cell. Proteomics.

[63] Wright J.J., Salvadori E., Bridges H.R., Hirst J., Roessler M.M. (2016). Small-volume potentiometric titrations: EPR investigations of Fe-S cluster N2 in mitochondrial complex I. J. Inorg. Biochem..

[64] Stoll S., Schweiger A. (2006). EasySpin, a comprehensive software package for spectral simulation and analysis in EPR. J. Magn. Reson..

[65] Blaza J.N., Vinothkumar K.R., Hirst J. (2018). Structure of the deactive state of mammalian respiratory complex I. Structure.

[66] Zivanov J., Nakane T., Scheres S.H.W. (2019). A Bayesian approach to beam-induced motion correction in cryo-EM single-particle analysis. IUCrJ.

[67] Zheng S.Q., Palovcak E., Armache J.P., Verba K.A., Cheng Y., Agard D.A. (2017). MotionCor2: Anisotropic correction of beam-induced motion for improved cryo-electron microscopy. Nat. Methods.

[68] Zhang K. (2016). Gctf: Real-time CTF determination and correction. J. Struct. Biol..

[69] Vilas J.L., Gómez-Blanco J., Conesa P., Melero R., Miguel de la Rosa-Trevín J., Otón J., Cuenca J., Marabini R., Carazo J.M., Vargas J., Sorzano C.O.S. (2018). MonoRes: Automatic and accurate estimation of local resolution for electron microscopy maps. Structure.

[70] Ramírez-Aportela E., Vilas J.L., Glukhova A., Melero R., Conesa P., Martínez M., Maluenda D., Mota J., Jiménez A., Vargas J., Marabini R., Sexton P.M., Carazo J.M., Sorzano C.O.S. (2020). Automatic local resolution-based sharpening of cryo-EM maps. Bioinformatics.

[71] de la Rosa-Trevín J.M., Quintana A., del Cano L., Zaldívar A., Foche I., Gutiérrez J., Gómez-Blanco J., Burguet-Castell J., Cuenca-Alba J., Abrishami V., Vargas J., Otón J., Sharov G., Vilas J.L., Navas J. (2016). Scipion: A software framework toward integration, reproducibility and validation in 3D electron microscopy. J. Struct. Biol..

[72] Amunts A., Brown A., Bai X.-C., Llacer J.L., Hussain T., Emsley P., Long F., Murshudov G., Scheres S.H.W., Ramakrishnan V. (2014). Structure of the yeast mitochondrial large ribosomal subunit. Science.

[73] Afonine P.V., Klaholz B.P., Moriarty N.W., Poon B.K., Sobolev O.V., Terwilliger T.C., Adams P.D., Urzhumtsev A. (2018). New tools for the analysis and validation of cryo-EM maps and atomic models. Acta Crystallogr. D Struct. Biol..

[74] Angerer H., Zwicker K., Wumaier Z., Sokolova L., Heide H., Steger M., Kaiser S., Nübel E., Brutschy B., Radermacher M., Brandt U., Zickermann V. (2011). A scaffold of accessory subunits links the peripheral arm and the distal proton-pumping module of mitochondrial complex I. Biochem. J..

[75] Abdrakhmanova A., Zickermann V., Bostina M., Radermacher M., Schägger H., Kerscher S., Brandt U. (2004). Subunit composition of mitochondrial complex I from the yeast *Yarrowia lipolytica*. Biochim. Biophys. Acta.

