# Supplementary material for: A conserved arginine residue is critical for stabilizing the N2 FeS cluster in mitochondrial complex I
Source: J Biol Chem. 2021 Feb 26;296:100474. doi: 10.1016/j.jbc.2021.100474 (PMC8042128; doi:10.1016/j.jbc.2021.100474)
Supplement: Supplemental Figures S1–S4 and Tables S1–S2 [file mmc1.pdf]

Supporting Information

A conserved arginine residue is critical for stabilizing the N2 FeS cluster in mitochondrial complex I

Mikhail A. Hameedi[1], Daniel N. Grba[1], Katherine H. Richardson[2,3], Andrew J. Y. Jones[1], Wei Song[2], Maxie M. Roessler[2,3*], John J. Wright[1,2*] & Judy Hirst[1*]

[1] Medical Research Council Mitochondrial Biology Unit, University of Cambridge, Cambridge CB2 0XY, UK
[2]School of Biological and Chemical Sciences, Queen Mary University of London, Mile End Road, London E1 4NS, UK.
[3]Department of Chemistry, Imperial College London, Molecular Sciences Research Hub, White City Campus, Wood Lane, London W12 0BZ, UK.

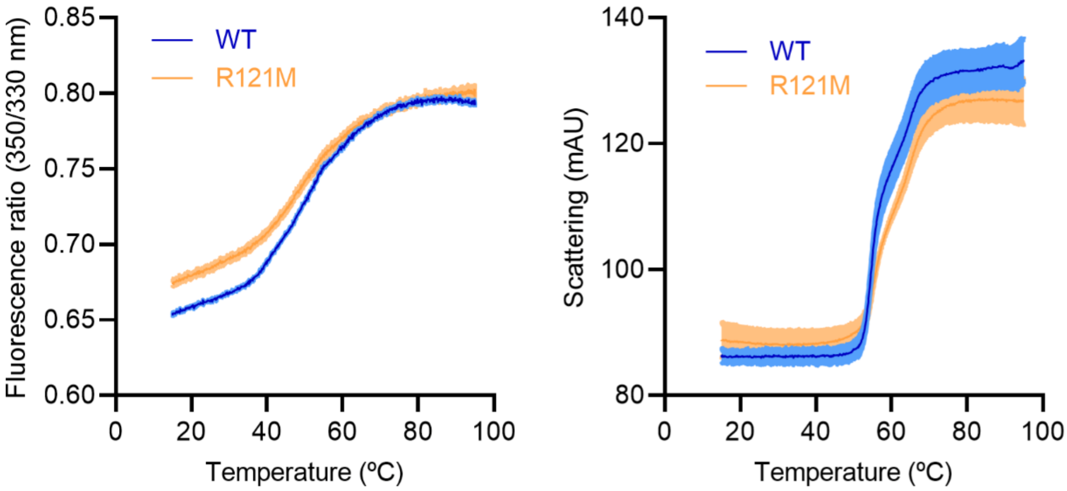

Figure S1. Differential scanning fluorimetry traces for wild-type and R121M complex I. Melting temperature (T_m) and aggregation temperature (T_{agg}) were measured for 1 mg mL^{-1} of DDM purified complex I. Traces are shown as the mean (± SEM) of 3 independent measurements. See Experimental Procedures for assay details. The T_{agg} temperature was taken as the average of the two inflection points from the DSF traces, which appeared at consistent temperatures in both the wild-type and R121M complex I.

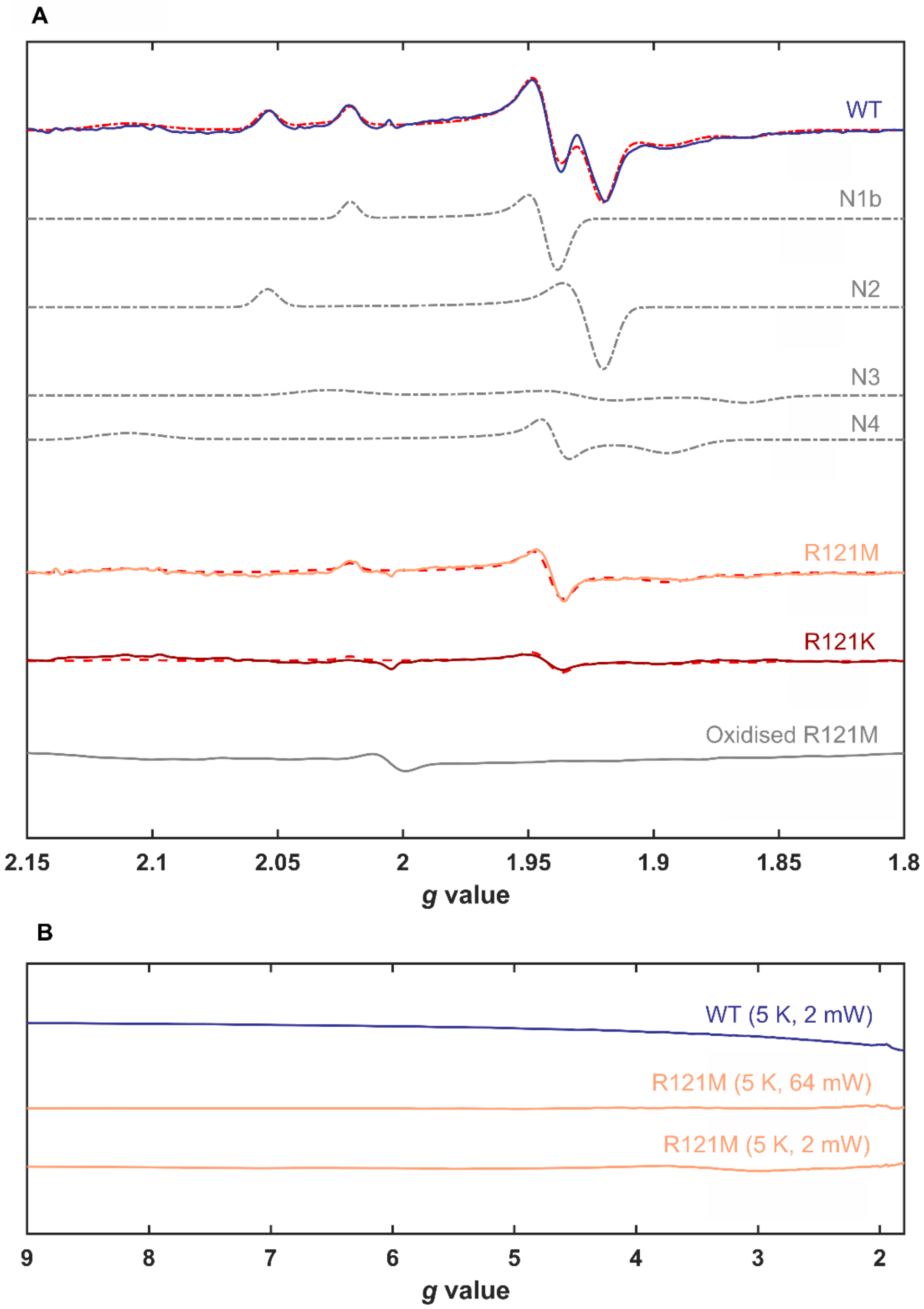

Figure S2. X-band CW EPR spectra of wild-type and mutant complex I. (A) Reduced WT (30 µM), R121M (12 µM), R121K (6 µM) and oxidised R121M in grey spectra at 15 K, 2 mW. Simulations in dotted lines, individual clusters in grey, sum of clusters overlaid in red. WT was simulated with 23% N1, 32% N2, 17% N3, 28% N4 (0.72:1:0.53:0.88). R121M and R121K spectra simulated using the WT fit scaled to protein concentration with zero contribution from N2. The g = 2.003 signal for the species present in the oxidised R121M spectrum originates from excess of oxidising agent (potassium ferricyanide). (B) Wide field sweep spectra of reduced WT and R121M at indicated temperatures and microwave powers in blue and orange respectively.

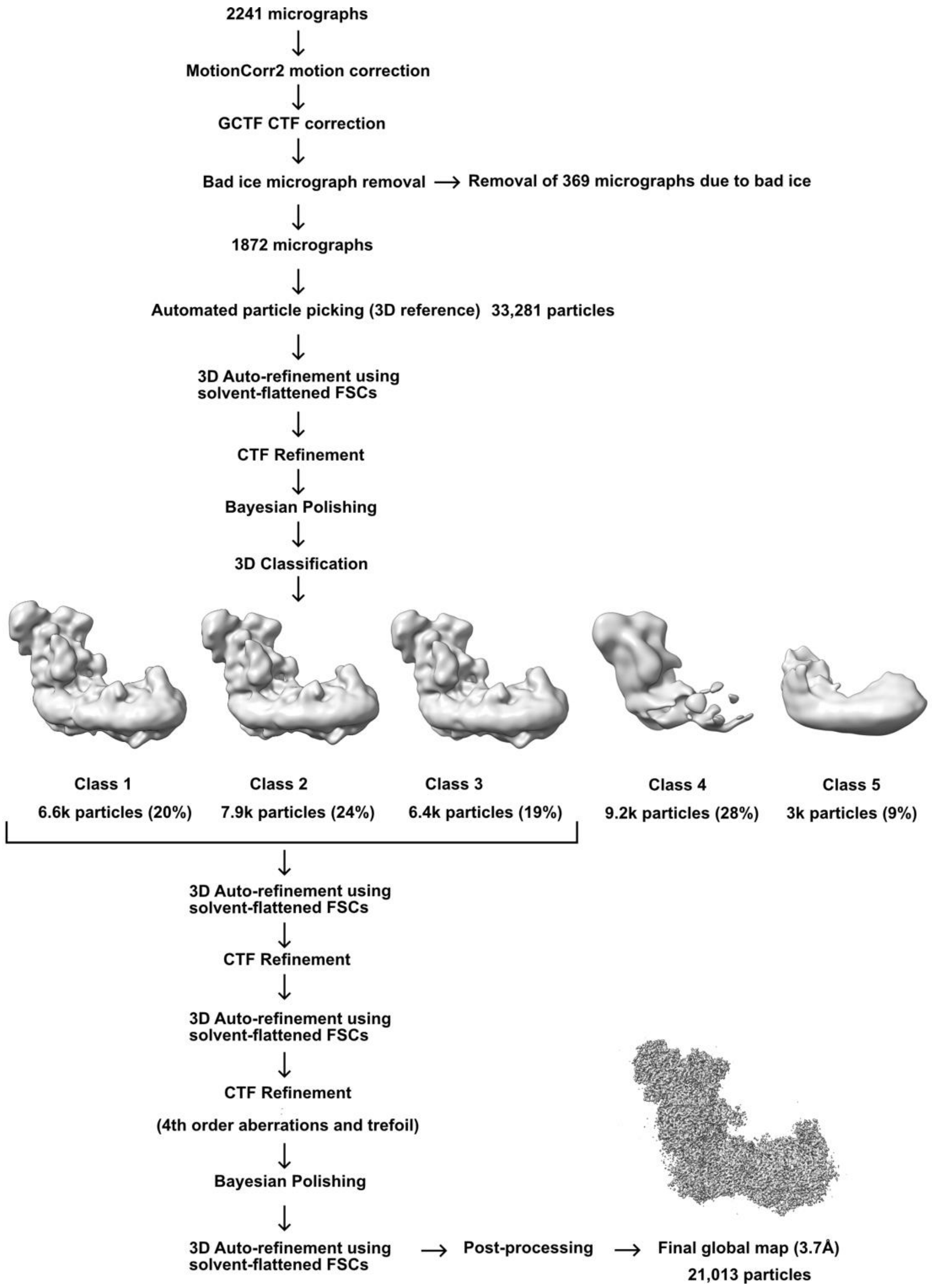

Figure S3. The classification and refinement scheme of the single-particle cryo-EM density map for the NDUFS2-R121M mutant of *Y. lipolytica* complex I. The workflow was implemented in RELION 3.1 (45, 46) and the final 3.7 Å resolution was achieved with 21,013 particles. See Experimental Procedures for further details.

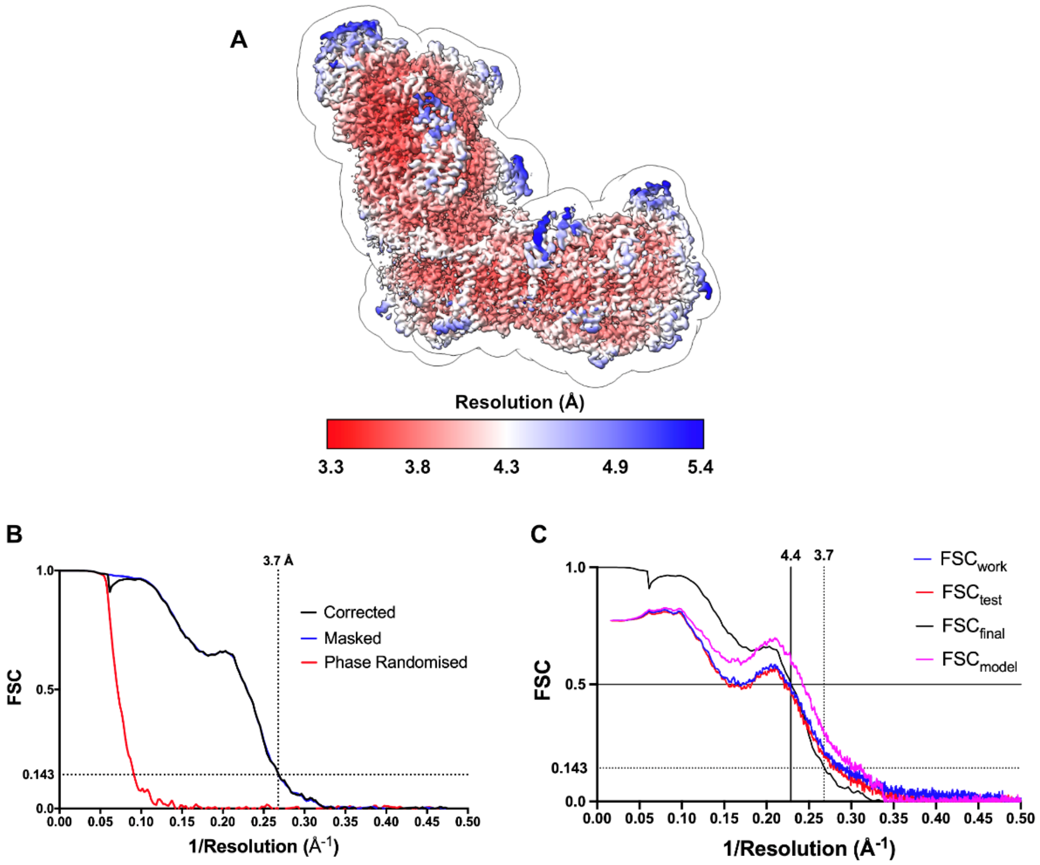

Figure S4. Analysis of the structure of R121M complex I. (A) Local resolution estimation of the R121M complex I density map and the global resolution estimate of 3.7 Å at FSC = 0.143 from the masked Fourier shell correlation curves. The mask used is indicated by the black outline. (B) FSC curves for the masked and corrected outputs from RELION. The phase randomised (red) line drops to zero rapidly indicating the mask used is not too tight. (C) To test whether the R121M model is overfitted (72, 73) to the map, model atoms were displaced by 0.5 Å, and refined in one half map, followed by FSC map–model validation (FSC_{work}). The output model was then FSC map–model validated against the other independent half map (FSC_{test}). The model curves are consistent beyond the 3.7 Å used for refinement and show little evidence of overfitting, with the small separation likely due to the original model coming from the near-complete 2.7 Å structure. No residues beyond the disruption of N2 were removed as these regions are very unlikely to differ from in the wild-type enzyme.

Table S1. Cryo-EM data collection parameters, refinement and validation statistics

	Y. lipolytica NDUFS2-R121M complex I (EMD-11969, PDB 7B0N)
**Data collection and processing**	
Magnification	130,000×
Voltage (kV)	300
Electron exposure ($e^{–}/Å^2$)	47.9
Defocus range (μm)	-1.5 to -2.7
Pixel size (Å)	1.07
Symmetry imposed	*C*1
Initial particle images (no.)	33,281
Final particle images (no.)	21,013
Map resolution (Å)	3.7 (4.4)
FSC threshold	0.143 (0.5)
Map resolution range (Å)	3.3–8.1
**Refinement**	
Initial model used	PDB 6YJ4
Model resolution (Å)	3.3 (4.1)
FSC threshold	0.143 (0.5)
Model resolution range (Å)	472.5–4.1
Map sharpening *B* factor ($Å^2$)	-62.8
Model composition	
Non-hydrogen atoms	65350
Protein residues	8094
Ligands	36
B factors ($Å^2$)	
Protein	37.85
Ligand	39.82
R.m.s. deviations	
Bond lengths (Å)	0.014
Bond angles (°)	1.446
**Validation**	
MolProbity score	1.38
Clashscore	2.24
Poor rotamers (%)	0
Ramachandran plot	
Favored (%)	94.38
Allowed (%)	5.52
Disallowed (%)	0.10

Table S2. NUCM-R121M *Y. lipolytica* complex I subunit model summary.

Subunit	*Y. lipolytica* nomenclature	Chain	Total residues (mature protein numbering)	Residues modelled [%] (range built)	Residues without side chains	Modelled with side chains [%]
NDUFS1	NUAM	G	694 (35–728)	100 (35–728)	0	100
NDUFV1	NUBM	F	470 (19–488)	98 (29–488)	0	100
NDUFS2	NUCM	D	444 (23–466)	98 (30–466)	90–91, 95–97, 121, 144	98
NDUFS3	NUGM	C	263[a] (31–281)	92 (33–274)	0	100
NDUFV2	NUHM	E	216 (28–243)	100 (28–243)	0	100
NDUFS8	NUIM	I	198 (32–229)	96 (39–229)	0	100
NDUFS7	NUKM	B	183 (28–210)	97 (34–210)	85, 86	99
NU1M	NU1M	H	341 (1–341)	100 (1–341)	210–219	97
NU2M	NU2M	N	469 (1–469)	100 (1–469)	0	100
NU3M	NU3M	A	128 (1–128)	73 (1–14, 49–128)	0	100
NU4M	NU4M	M	486 (1–486)	100 (1–486)	0	100
NU5M	NU5M	L	655 (1–655)	100 (1–655)	609–612	99
NU6M	NU6M	J	185 (1–185)	100 (1–185)	0	100
NULM	NULM	K	89 (1–89)	100 (1–89)	0	100
NDUFA9	NUEM	P	355 (21–375)	100 (21–375)	353–364	97
NDUFA5	NUFM	V	136 (9–144)	93 (19–144)	0	100
NDUFS6	NUMM	R	118 (19–136)	100 (19–136)	0	100
NDUFS4	NUYM	Q	137 (25–161)	91 (37–161)	0	100
NDUFA7	NUZM	c	182 (1–182)	100 (1–182)	0	100
NDUFA12	N7BM	f	137 (2–138)	100 (2–138)	0	100
NDUFA6	NB4M	W	123 (2–124)	100 (2–124)	0	100
NDUFAB1	ACPM1	T	84 (26–109)	96 (29–109)	0	100
NDUFA2	NI8M	S	86 (2–87)	100 (2–87)	0	100
NDUFA8	NUPM	X	171 (2–172)	100 (2–172)	0	100
NDUFA11	NUJM	Y	185 (14–198)	97 (20–198)	0	100
NDUFA13	NB6M	Z	122 (2–123)	100 (2–123)	0	100
NDUFS5	NIPM	e	88 (2–89)	77 (2–69)	0	100
NUXM*	NUXM	O	168 (2–169)	100 (2–169)	0	100
NDUFA3	NI9M	b	78 (1–78)	100 (1–78)	0	100

NDUFA1	NIMM	a	86<br>(2–87)	100<br>(2–87)	0	100
NDUFC2	NEBM	d	73<br>(2–74)	92<br>(2–68)	0	0
NDUFB11	NESM	g	204<br>(46–249)	97<br>(46–243)	75–85	94
NDUFB8	NIAM	l	125<br>(25–149)	100<br>(25–149)	0	100
NDUFB5	NUNM	h	119<br>(32–150)	95<br>(38–150)	0	100
NDUFB3	NB2M	k	59<br>(2–60)	80<br>(2–48)	0	100
NDUFB4	NB5M	m	92<br>(2–93)	100<br>(2–93)	0	100
NDUFB7	NB8M	o	98<br>(2–98)	85<br>(2–84)	0	100
NDUFAB1	ACPM2	U	88<br>(45–132)	100<br>(45–132)	0	100
NDUFB10	NIDM	p	91<br>(2–92)	100<br>(2–92)	0	100
NDUFB9	NI2M	n	108<br>(2–109)	100<br>(2–109)	0	100
NDUFB2	NIGM	j	67[b]<br>(1–67)	79<br>(7–59)	0	100
NDUFB6	NUUM	i	89<br>(2–90)	97<br>(2–87)	0	100

[a]Includes the hexa-Ala-hexa-His tag; [b]The mature protein predicted by Parey and coworkers (20); MTS-cleavage sites were assumed from previously published information (74, 75). *NUXM is referred to by *Y. lipolytica* nomenclature due to the absence of a homologue in the human enzyme.
